# Reinforcing the Research Coproduction Impact Pathway: A Response to Recent Commentaries

**DOI:** 10.34172/ijhpm.9302

**Published:** 2025-07-28

**Authors:** Chris McCutcheon, Ian D. Graham, Anita Kothari, Jo Rycroft-Malone

**Affiliations:** ^1^Centre for Practice-Changing Research, Ottawa Hospital Research Institute, Ottawa, ON, Canada.; ^2^School of Epidemiology and Public Health, University of Ottawa, Ottawa, ON, Canada.; ^3^School of Health Studies, Faculty of Health Sciences, University of Western Ontario, London, ON, Canada.; ^4^Faculty of Health and Medicine, Lancaster University, Lancaster, UK.

## Background

 Last year we published an editorial as an addendum to our book, *Research Coproduction in Healthcare,*^1,2^ which led to six thought provoking commentaries.^3-8^ These authors challenged us to reflect critically on our positions and embrace different perspectives, and we have tried to do so in this response. There were three prominent themes in our editorial that resonated with the commentators and are worth reiterating here. First, research coproduction needs authentic partnership to be effective, and this in turn requires “a principle-based and explicitly values-driven approach in which the skills, processes and attitudes required to nurture relationships between knowledge users and researchers are as important as the scientific approach itself” (p. 2).^1^ Second, principles and values on their own are insufficient: nurturing relationships, especially as partnerships become more complex, including more knowledge users from varied contexts and backgrounds, involves taking steps to redress power imbalances and inequities. Third, changes need to be made at all levels of the research system architecture to “provide the structures and resources for optimal partnership working” (p. 2).^1^

## An Underused Pathway, Not the Only Way

 In calling research coproduction “an underused pathway to impact” (p. 1),^1^ we assert that not enough research is produced for and with knowledge users, that a deficit in partnered research contributes to a deficit in evidence use. However, we agree with Estabrooks^8^ that research coproduction is not a panacea, nor is it the only way that research achieves impact. It is a good reminder that we should be cautious about the claims made about research coproduction. As Ramage et al^3^ and Cameron and Fiolet^5^ note, there remains a dearth of evidence about the effectiveness of this approach and filling this gap should be a priority for our field.

 Just as there are many ways to achieve impact, research coproduction varies according to its goals and scale. Bandola-Gill and colleagues^9^ identify five approaches in their synthesis of the literature: coproduction as science-politics relationships, knowledge democracy, transdisciplinary research, boundary management, and evidence use intervention. Our work has mainly intersected with the latter, which is a more instrumental approach in contrast to the transformational goals reflected in the research described by some commentators.^4,5,7^ The differences in our perspectives are perhaps reflections of diverse traditions and approaches, rather than fundamental points of disagreement.

## Power in Relationships

 Trusting relationships begin with the shared values and principles that are important to everyone on the team and ultimately become reflected in how they work together. Masterson and Laidlaw^6^ affirm this perspective and present a tool for helping teams to operationalize their shared values and principles. Ramage et al^3^ also note that coproduction is grounded in relationship building, highlighting the importance of emotional intelligence and interpersonal skills, particularly when interacting with individuals who may have communication impairments. Fundamentally, research coproduction is about relationships and trust, and like every human relationship, requires hard work to initiate and maintain. As Cameron and Fiolet^5^ mention, conflict is also inherent in research partnerships and can arise for different reasons, such as differing expectations, communication errors and power imbalances. However, when a relationship is strong and trusting, conflicts can often be resolved through frank and, occasionally, uncomfortable discussion.

 Conflict raises the issue of power, which is emerging as a critical concept in coproduction.^10^ In our editorial, most of our focus was on power sharing within a research coproduction team, operationalized as shared decision-making. For us, coproduction reflects the *collaborate* or partner level in engagement frameworks such as the International Association of Public Participation’s (IAP2) Spectrum of Public Participation.^11^ All team members are considered equal in terms of their expertise, and the team makes decisions collectively. As Cameron and Fiolet^5^ note, working towards power sharing does not preclude coproduction teams from also working towards equity.

 Conte and Zapata^7^ go further and make a compelling case for a paradigm shift from equality-driven coproduction to equity-driven coproduction. This entails redistributing power to equity-deserving research partners to support their self-determination. We argue this approach is reflected by the level of *empowerment* on the IAP2 spectrum of participation, which by definition becomes community-led rather than coproduced.^11^ We concur there is a growing need for research that promotes self-determination of many groups in society, which means deconstructing, decolonizing and re-purposing standard research practices. However, we assert it may be preferable to differentiate a community-led approach from a coproduction approach.

## Underused and Under-Supported

 Cameron and Fiolet^5^ challenged our suggestion of “systematizing” research coproduction, which implied an intent to standardize if not colonize research practices that should remain open and adaptable. We agree that research coproduction should not be regimented. A principles-based approach can describe what makes coproduction teams effective and steer those new to the practice away from any misapprehension that it is a method. The term “systematizing” reflects our view that we need a systems-oriented approach to embed research coproduction into all levels of research support, or a re-tooling of these supports so that instead of working in spite of the system, research coproduction is “business as usual”^1^ (p. 2).

 Whilst regimentation should be avoided, guidance could contribute to the cohesion of research coproduction. Masterson and Laidlaw’s^6^ Co-MPASS (Co-producing Meaningful Principles and Sharing Standards) framework is an excellent example of how to focus guidance on the principles and values of true partnerships. Going further, we support Ramage and colleagues’^3^ suggestion about the potential for reporting guidance. Although we are a community that values sharing, we tend to remain fastidious about describing our research methods but divulge little about how we work with our partners. Principle-based reporting guidance may help with transparency and share learning.

## Conclusion

 Whilst research coproduction is not a panacea, our theory remains that research which is authentically and judiciously coproduced has the greatest potential for impact. We encourage a continued debate about principle-based research coproduction – in this way we will learn from each other and further develop the evidence base.

## Acknowledgements

 We thank Madison Roche for support in formatting the manuscript.

## Ethical issues

 Not applicable.

## Conflicts of interest

 Authors declare that they have no conflicts of interest.
